# Supplementary material for: Estimating the contribution of setting-specific contacts to SARS-CoV-2 transmission using digital contact tracing data
Source: Nat Commun. 2024 Jul 19;15:6103. doi: 10.1038/s41467-024-50487-7 (PMC11271501; doi:10.1038/s41467-024-50487-7)
Supplement: Supplementary file 1 — Supplementary Information [file 41467_2024_50487_MOESM1_ESM.pdf]

Supplementary Material for

Estimating the contribution of setting-specific contacts to SARS-CoV-2 transmission using digital contact tracing data

Authors: Zengmiao Wang[1]†, Peng Yang[2,3]†, Ruixue Wang[1]†, Luca Ferretti[4,5], Lele Zhao[4,5], Shan Pei[1], Xiaoli Wang[2,3], Lei Jia[2,3], Daitao Zhang[2,3], Yonghong Liu[2,3], Ziyan Liu[1], Quanyi Wang[2,3]*, Christophe Fraser[4,5], Huaiyu Tian[1]*

Affiliations:

[1]State Key Laboratory of Remote Sensing Science, Center for Global Change and Public Health, Faculty of Geographical Science, Beijing Normal University, Beijing, China

[2]Beijing Center for Disease Prevention and Control, Beijing, China

[3]Beijing Research Center for Respiratory Infectious Diseases, Beijing, China

[4]Pandemic Sciences Institute, Nuffield Department for Medicine, University of Oxford, Oxford, UK

[5]Big Data Institute, Li Ka Shing Centre for Health Information and Discovery, Nuffield, Department for Medicine, University of Oxford, Oxford, UK

*Correspondence and requests for materials should be addressed to H.T. (tianhuaiyu@gmail.com) or Q.W. (wangqy@bjcdc.org)

†These authors contributed equally to this work

The file includes:

Figures S1 to S8

Tables S1 to S6

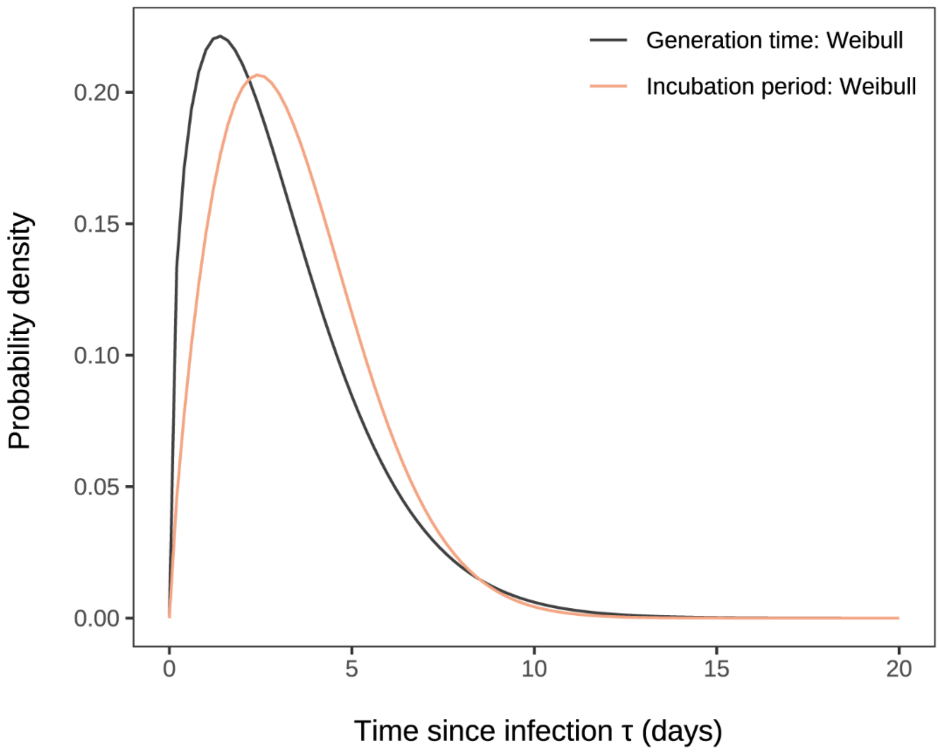

Figure S1. The estimated density function for generation time and incubation period.

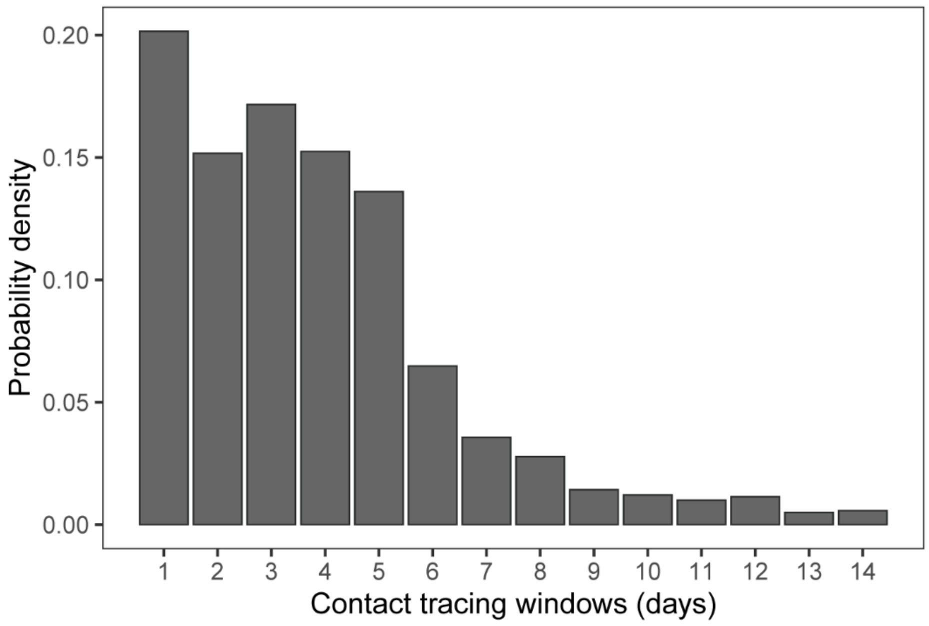

Figure S2. The distribution of contact tracing window defined in our dataset.

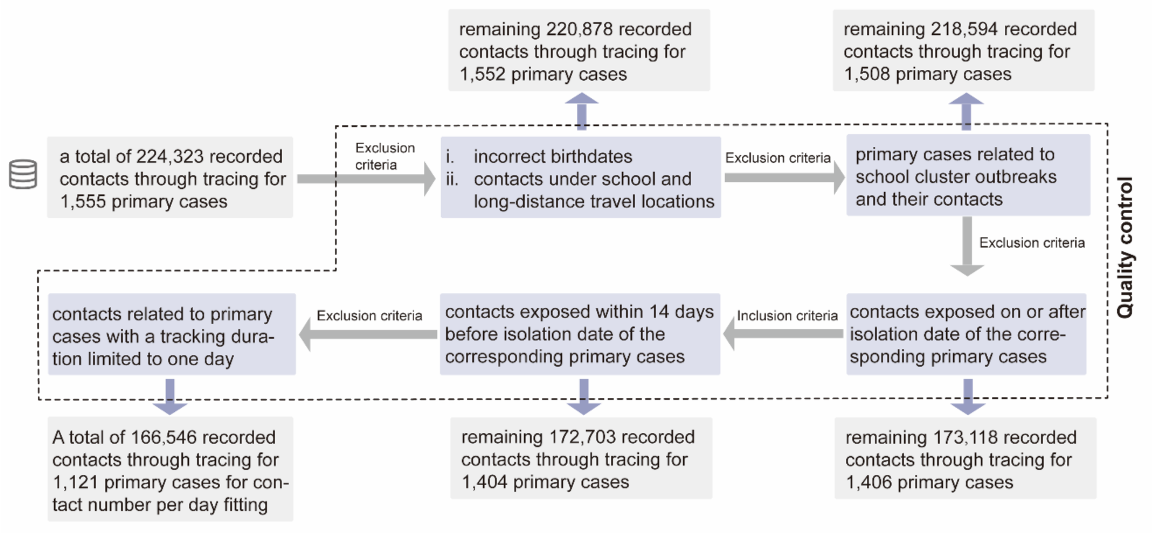

Figure S3. Quality control for the contact data.

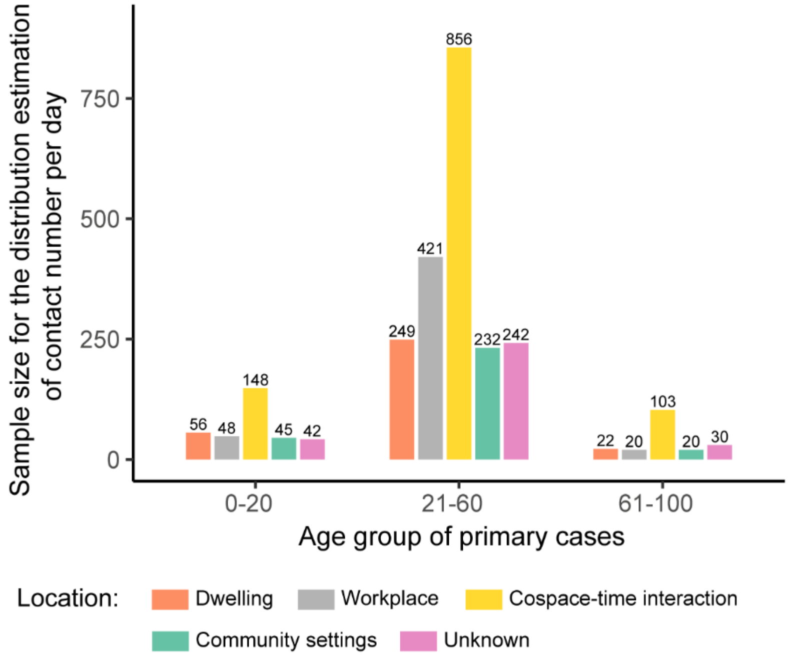

Figure S4. Sample size for the stratification based on age and location for contact number per day.

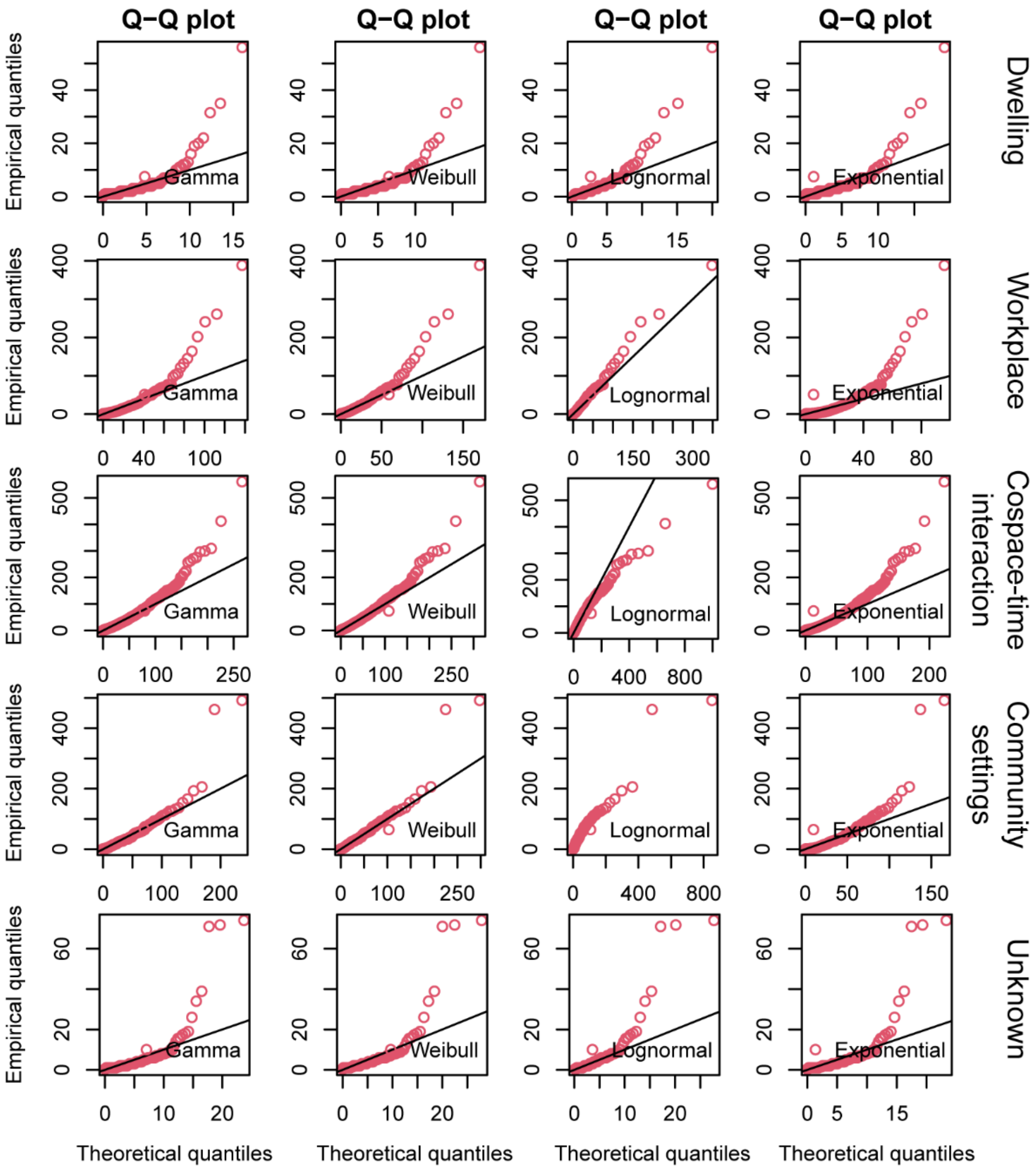

Figure S5. Quantile-quantile (Q-Q) plots for the estimated distribution for contact number per day under different locations.

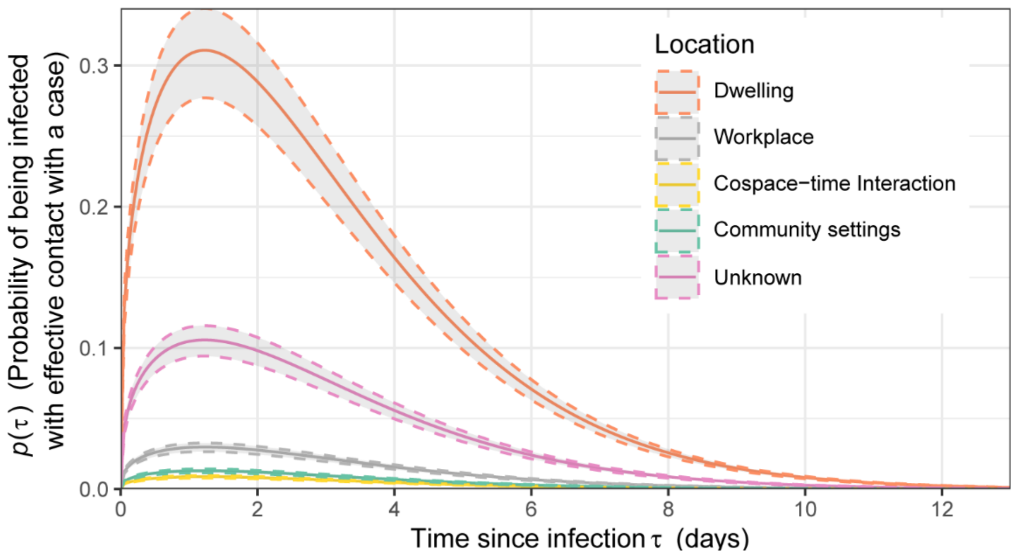

Figure S6. Dynamic infectiousness under different locations. The probability of being infected with effective contact under different locations with a case (p is shown as a function of the time since infection τ). Distinct colors represent the median of calculated p for the five different locations. The gray area represents the 95% confidence interval.

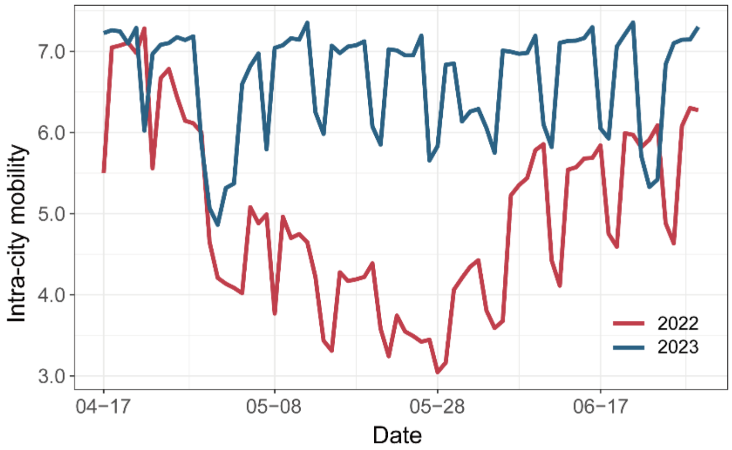

Figure S7. The comparison of intra-city mobility between 2022 and 2023 in Beijing. The mobility index in 2023 (after China's reopening) was, on average, 1.34 times higher than that in 2022.

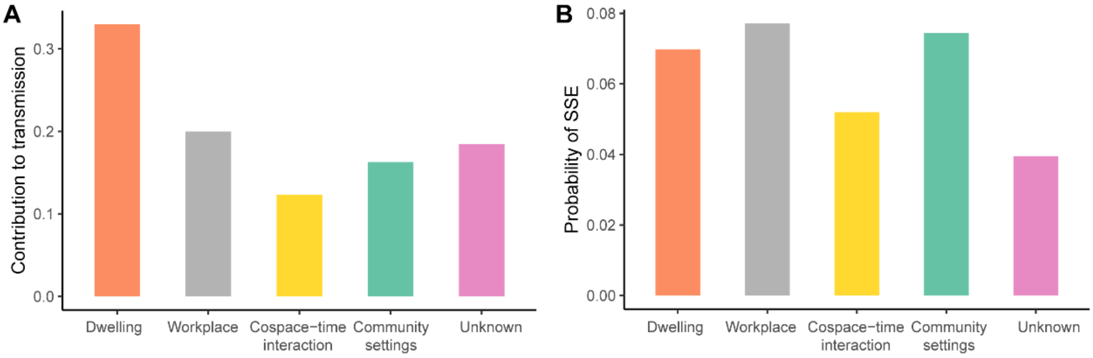

Figure S8. The transmission contribution and the super-spreading event risk under different locations after adjusting the Dynamic zero COVID policy based on the intra-city mobility. The impact of social distance policy on contact patterns was assumed to be proportional to intra-city mobility. Consequently, we multiplied the mean contacts for workplaces, cospace-time interactions, community settings, and unknown settings by 1.34 (Figure S7). The contact pattern for dwellings was not adjusted since these contacts are essential and less likely to be affected by control measures. No statistics were derived due to the use of a single sample for each bar.

Table S1. The definition of contacts with infected individuals under different locations.

**Location**	**Definition**
Dwelling	a contact who resides with or provides care for an individual infected with SARS-CoV-2.
Workplace	a colleague sharing a workspace with an individual infected with SARS-CoV-2.
Cospace-time interaction	meeting the following two conditions: (1) individuals who have been present during the same time period as the case, or within the subsequent three hours, and without direct close contact; (2) individuals who have been in poorly ventilated and confined spaces, particularly those with a per capita area below 1.5 m^2 (such as participating in collective entertainment or fitness endeavors, companions or acquaintances sharing meals at bars or restaurants; social participants sharing the same car-hailing service and individuals sharing elevators).
Community settings	a contact who lives in the same residential structure, or acts as a neighbor to an individual infected with SARS-CoV-2.
Unknown settings	special scenarios where it's difficult to determine categories (such as those encountered on crowded streets).

Table S2. Summary statistics of SARS-CoV-2 cases in the Omicron outbreak in Beijing.

**Period**	April 17, 2022 to June 29, 2022
**Number of cases**	2,230
**Clinical outcome (%):**	
Symptomatic	1,787 (80.1%)
Asymptomatic	443 (19.9%)
**Gender (%)**	
Male	1,226 (55.0%)
Female	1,004 (45.0%)
**Age group (%)**	
1-20 years old	408 (18.3%)
21-60 years old	1,557 (69.8%)
≥61 years old	265 (11.9%)
**Vaccination status at the end of the period (%):**	
1 dose	70 (3.1%)
2 doses	516 (23.1%)
3 doses	1,397 (62.6%)
4 doses	1 (0.1%)
none	246 (11.1%)

Table S3. Summary statistics of contacts in the Omicron outbreak in Beijing.

**Number of contacts**	220,878*
**Number of primary cases (with recorded contacts)**	1,552
**Number of transmission events**	1,495
**Number of infectors from transmission events**	451
**Number of infectees (contacts being positive) from transmission events (%)**	1,495/220,878 (0.7%)
**Age group of contacts being positive (%)**	
1-20 years old	297/19,698 (1.5%)
21-60 years old	1,078/181,924 (0.6%)
≥61 years old	172/19,256 (0.9%)
**Number of contacts being positive (%) under different locations**	
Dwelling	383/3,024 (12.7%)
Workplace	234/19,279 (1.2%)
Cospace-time interaction	603/167,713 (0.4%)
Community settings	141/26,541 (0.5%)
Unknown settings	186/4,321 (4.3%)

*: after removing the missing values and contacts under location of school and long-distance traveling, 220,878 contacts were left. Please see the section of Quality control for the contacts for more details.

Table S4. The estimations for incubation period and generation time under different forms of distributions in Beijing during the Spring of 2022, China.

Epidemiological feature	Distributions		Estimate (95%CI)		LOO IC$
	**Type of distribution**	**Sample size**	**Mean (days)**	**SD (days)**	
Generation time	Weibull	n = 48	2.99 (2.44, 3.75)	2.17 (1.68, 3.10)	197.4
	Gamma		0.62 (0.28, 1.24)	0.53 (0.28, 1.91)	205.2
	Lognormal		8.69 (4.48, 23.61)	37.32 (12.40, 226.22)	260.3
	Calculation from the data		3.00	1.83	-
Incubation period	Weibull	n = 226	3.46 (3.21, 3.73)	2.01 (1.82, 2.45)	935.6
	Gamma		1.25 (0.87, 1.77)	0.86 (0.65, 1.13)	976.7
	Lognormal		6.17 (5.01, 7.88)	12.56 (8.88, 19.10)	1224.9
	Calculation from the data		3.53	1.83	-

$LOO IC indicates the goodness-of-fit, where lower values indicate a better fit and differences larger than two are statistically relevant

Table S5. Comparison between the age demographics in Beijing with the age distribution of cases and contacts in the Omicron outbreak in Beijing. The data presented in this table is consistent with that in Figure 2.

Age Group	Age demographics in Beijing in 2020	Age distribution for the cases with identified contacts	Age distribution for contacts
1-20 years old	14.6%	13.4% (150/1,121)	7.8% (12,909/166,546)
21-60 years old	65.2%	77.3% (866/1,121)	84.3% (140,409/166,546)
≥61 years old	20.2%	9.3% (105/1,121)	7.9% (13,228/166,546)

Table S6. The sensitivity analysis for the contact number per day of tracing when aligning the cases' age distribution with the age demographics in Beijing. The cases were sub-sampled and fitted to Gamma distribution. The mean and variance were obtained from the fitted Gamma distribution, and this process was repeated for 100 times. The median and 95% confidence interval (CI) were then derived from the sub-sampling results.

Location	Median (95% CI) for the mean of Gamma distribution	Median (95% CI) for the variance of Gamma distribution
**Dwelling**	2.94 (2.45-3.46)	6.67 (3.31-10.79)
**Workplace**	13.27 (10.42-16.74)	305.40 (160.97-558.70)
**Cospace-time interaction**	28.35 (25.39-31.01)	1058.36 (803.71-1324.53)
**Community settings**	24.87 (19.62-31.29)	1053.42 (616.97-1820.25)
**Unknown settings**	3.64 (2.83-4.56)	13.94 (6.34-27.08)
